# Bis{*N*′-[3-(4-nitro­phen­yl)-1-phenyl­prop-2-en-1-yl­idene]-*N*-phenyl­carbamimido­thio­ato}zinc(II): crystal structure, Hirshfeld surface analysis and computational study

**DOI:** 10.1107/S2056989021007398

**Published:** 2021-07-27

**Authors:** Ming Yueh Tan, Huey Chong Kwong, Karen A. Crouse, Thahira B. S. A. Ravoof, Edward R. T. Tiekink

**Affiliations:** a Department of Physical Science, Faculty of Applied Sciences, Tunku Abdul Rahman University College, 50932 Setapak, Kuala Lumpur, Malaysia; b Research Centre for Crystalline Materials, School of Medical and Life Sciences, Sunway University, 47500 Bandar Sunway, Selangor Darul Ehsan, Malaysia; c Department of Chemistry, Faculty of Science, Universiti Putra Malaysia, UPM, Serdang 43400, Malaysia; d Department of Chemistry, St. Francis Xavier University, PO Box 5000, Antigonish, NS B2G 2W5, Canada; e Foundry of Reticular Materials for Sustainability (FORMS), Materials Synthesis and Characterization Laboratory, Institute of Advanced Technology, Universiti Putra Malaysia, 43400 Serdang, Selangor Darul, Ehsan, Malaysia

**Keywords:** crystal structure, zinc, Schiff base, thio­semicarbazone, hydrogen bonding, Hirshfeld surface analysis

## Abstract

The title zinc bis­(thio­semicarbazone) complex, [Zn(C_22_H_17_N_4_O_2_S)_2_], comprises two *N*,*S*-donor anions, leading to a distorted tetra­hedral N_2_S_2_ donor set. The resultant five-membered chelate rings are nearly planar and form a dihedral angle of 73.28 (3)°. The configurations about the endocyclic- and exocyclic-imine bonds are *Z* and *E*, respectively, and that about the ethyl­ene bond is *E*. The major differences in the conformations of the ligands are seen in the dihedral angles between the chelate ring and nitro­benzene rings [40.48 (6) *cf*. 13.18 (4)°] and the *N*-bound phenyl and nitro­benzene ring [43.23 (8) and 22.64 (4)°]. In the crystal, a linear supra­molecular chain along the *b*-axis direction features amine-N—H⋯O(nitro) hydrogen bonding. The chains assemble along the 2_1_-screw axis through a combination of phenyl-C—H⋯O(nitro) and π(chelate ring)–π(phen­yl) contacts. The double chains are linked into a three-dimensional architecture through phenyl-C—H⋯O(nitro) and nitro-O⋯π(phen­yl) inter­actions.

## Abstract 2

The N_2_S_2_ donor set about the zinc atom in the title complex has a geometry approaching tetra­hedral. A linear supra­molecular chain featuring amine-N—H⋯O(nitro) hydrogen bonding is noted in the crystal.

## Chemical context   

Thio­semicarbazones constitute part of the versatile nitro­gen- and sulfur-donor ligands important in coordination chemistry because of their variable donor properties, structural diversity and pharmacological applications. These ligands usually act as monodentate or bidentate ligands and coordinate with transition and non-transition metal ions either in neutral or anionic form through thione/thiol­ate-sulfur and azomethine/imine-nitro­gen donor atoms (Lobana *et al.*, 2009 ▸; Prajapati & Patel, 2019 ▸; Şen Yüksel, 2021 ▸). The pharmacological activities of metal complexes are usually enhanced compared to their parent free thio­semicarbazone ligands (Mathews & Kurup, 2021 ▸). The enhanced activities may be attributed to the redox potential and increased lipophilicity of the metal complexes (Rapheal *et al.*, 2021 ▸). Transition-metal complexes derived from thio­semicarbazones exhibit widespread pharmacological activities inclusive of anti-tubercular (Khan *et al.*, 2020 ▸), anti-microbial (Nibila *et al.*, 2021 ▸), anti-bacterial (Prajapati & Patel, 2019 ▸), anti-malarial (Savir *et al.*, 2020 ▸), anti-diabetic (Kumar *et al.*, 2020 ▸), anti-viral (Rogolino *et al.*, 2015 ▸) and anti-cancer (Anjum *et al.*, 2019 ▸; Balakrishnan *et al.*, 2019 ▸). In this work, 4-phenyl-3-thio­semicarbazide was condensed with 4-nitro­chalcone to form the thio­semicarbazone, which was then complexed with zinc(II) in a molar ratio of 2:1 to form the title compound, hereafter (I). In a continuation of on-going studies of metal complexes derived from thio­semicarbazones and their parent ligands (Tan, Ho *et al.*, 2020 ▸; Tan, Kwong *et al.* 2020*a* ▸,*b* ▸), herein the synthesis, structure determination, Hirshfeld surface analysis and computational chemistry of (I) are described. 
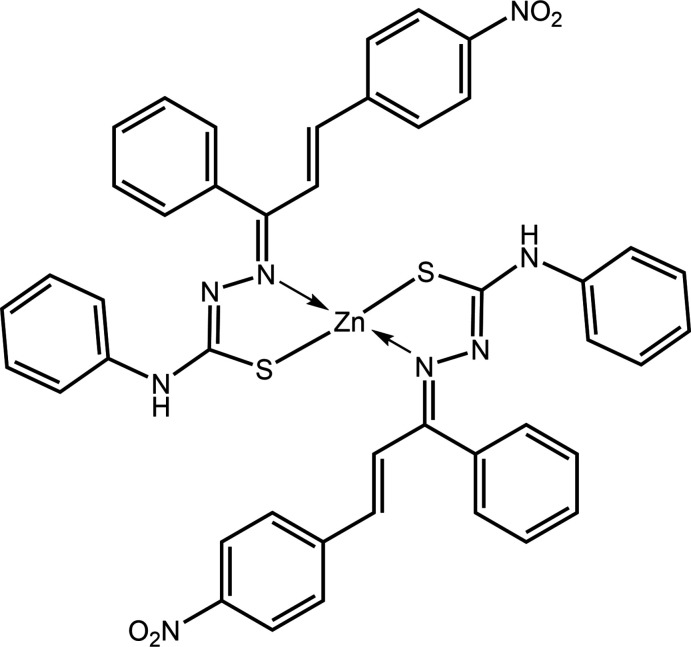


## Structural commentary   

**Figure 1:**
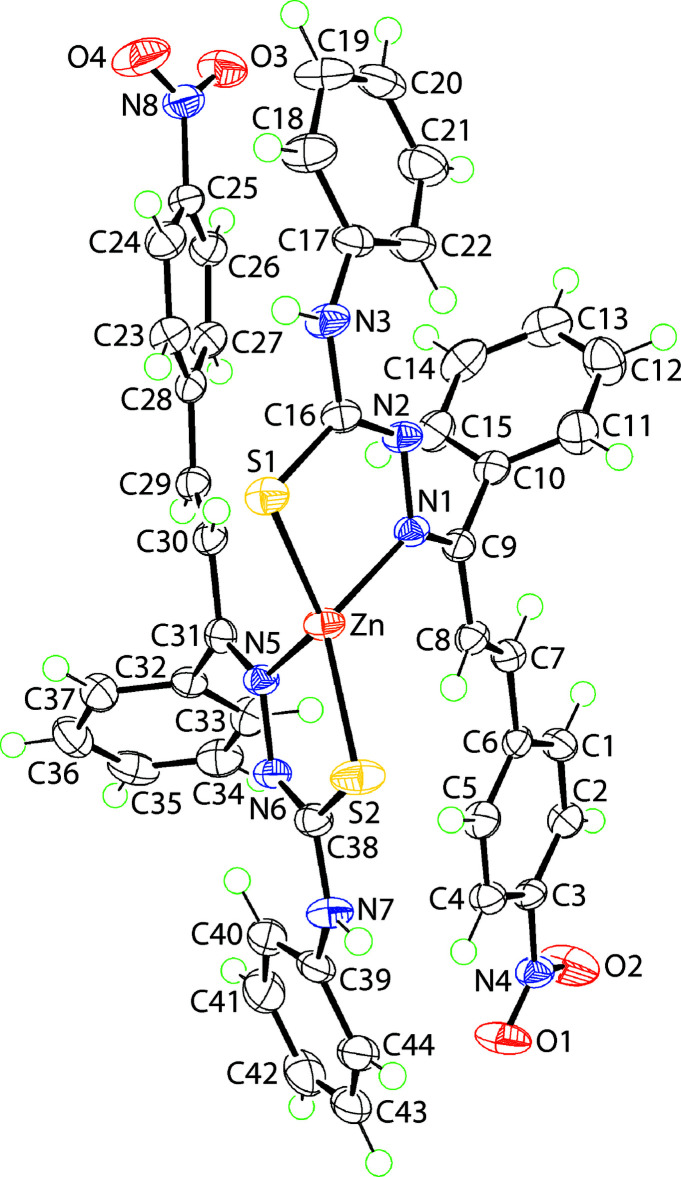
The mol­ecular structure of (I) showing the atom-labelling scheme and displacement ellipsoids at the 70% probability level.

The mol­ecular structure of (I), Fig. 1 ▸, comprises a zinc atom *S*,*N*-coordinated by two thio­semicarbazone anions within an N_2_S_2_-donor set. From the data in Table 1 ▸, the key geometric parameters for both ligands bear a close similarity. However, the Zn—S1 and Zn—N1 bond lengths are shorter and longer, respectively, compared with the Zn—S2 and Zn—N5 bonds, each by *ca* 0.07 Å. The angles about the zinc atom range from an acute 86.77 (4)° for the S1—Zn—N1 chelate angle, to a wide 131.16 (2)°, for S1—Zn—S2, consistent with an approximate tetra­hedral geometry. The mode of coordination of the thio­semicarbazone ligands leads to the formation of five-membered chelate rings. These are nearly planar with r.m.s. deviations of 0.0459 and 0.0152 Å for the S1- and S2-containing rings, respectively. However, the maximum deviation from the plane through the S1-chelate ring of −0.0613 (9) Å for the N1 atom suggests an alternate description of the conformation of the S1-ring might be valid. Another description might be an envelope conformation with the zinc atom lying 0.209 (3) Å out of the plane of the four remaining atoms (r.m.s. deviation = 0.0005 Å). The dihedral angle between the mean plane through the rings is 73.28 (3)°. There are three formal double bonds in each thio­semicarbazone anion. Owing to chelation, the configuration about the endocyclic imine bond is *Z* whereas that about the exocylic imine bond is *E*; the configuration of the ethyl­ene bond is *E*.

Some major differences are noted in the conformations of the ligands. Thus, the sequence of dihedral angles formed between the chelate ring and the imine-phenyl, *N*-bound phenyl and nitro­benzene rings is 72.41 (5), 16.96 (11) and 40.48 (6)°, respectively, for the S1-ring compared with 82.47 (6), 20.33 (5) and 13.18 (4)°, respectively, for the S2-ring. Similarly, the pairs of dihedral angles between the imine- and *N*-bound phenyl rings, *i.e*. 59.15 (6) and 76.48 (8)°, and *N*-bound phenyl and nitro­benzene rings, *i.e*. 43.23 (8) and 22.64 (4)°, show notable differences; the dihedral angles between the imine-phenyl and nitro­benzene rings are comparable, *i.e*. 82.28 (7) and 85.67 (7)°. Finally, the nitro groups present different relative orientations with respect to the benzene rings they are connected to, with the N4-nitro group being twisted out of the plane. This is shown in the value of the C2—C3—N4—O1 torsion angle of 161.88 (18)° compared with −0.4 (3)° for the C26—C25—N8—O3 torsion angle.

## Supra­molecular features   

**Figure 2:**
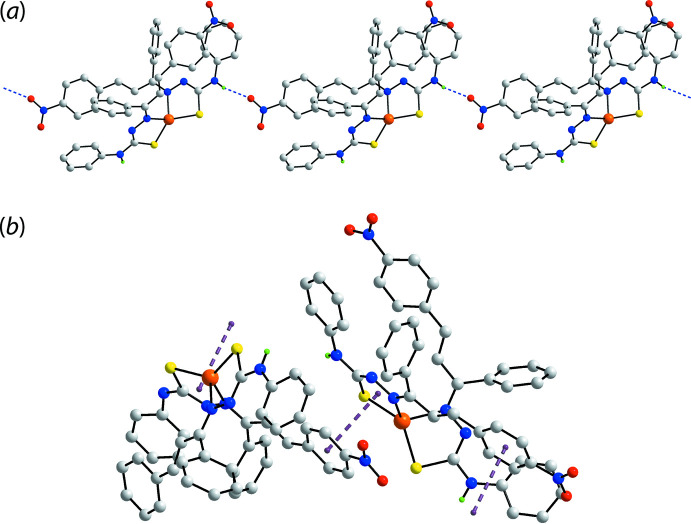
Mol­ecular packing in (I): (*a*) a view of the linear supra­molecular chain featuring amine-N—H⋯O(meth­oxy) hydrogen bonding shown as blue dashed lines and (*b*) detail of the π(phen­yl)–π(chelate ring) inter­action shown as purple dashed lines. In each image, non-participating H atoms are omitted.

**Figure 3:**
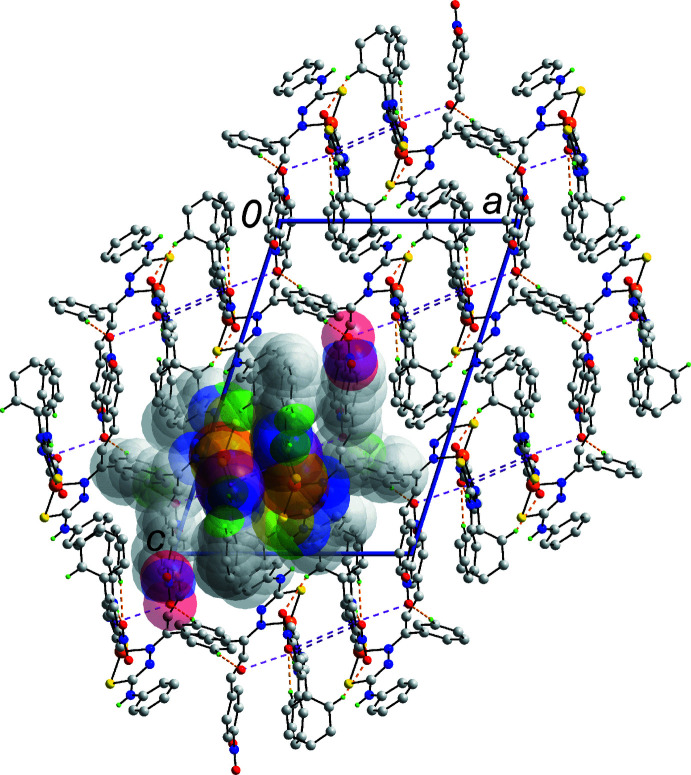
A view of the unit-cell contents shown in projection down the *b*-axis direction. The C—H⋯O, N—O⋯π and π–π inter­actions are shown as orange, pink and purple dashed lines, respectively. The non-participating H atoms are omitted and one chain sustained by amine-N—H⋯O(meth­oxy), π(phen­yl)–π(chelate ring) and phenyl-C—H⋯O4(nitro) inter­actions is highlighted in space-filling mode.

Conventional amine-N7—H⋯O4(nitro) hydrogen bonds are noted in the crystal of (I). These feature within a linear supra­molecular chain aligned along the *b*-axis direction, Table 2 ▸ and Fig. 2 ▸(*a*). The hydrogen bonds involve the N7-amine, there being no apparent role for the N3-amine in the supra­molecular aggregation. A phenyl-C44—H⋯O4(nitro) contact provides extra stability to the chain and indicates the nitro-O4 atom forms two contacts. Chains assemble about the 2_1_-screw axis *via* a combination of phenyl-C37—H⋯O3(nitro) and π–π contacts. The π–π contacts are of particular inter­est in that the participating rings are a phenyl and a chelate ring, as highlighted in Fig. 2 ▸(*b*); such inter­actions are now well recognized in the supra­molecular chemistry of metal complexes and impart significant energies of stabilization to the packing (Malenov *et al.*, 2017 ▸; Tiekink, 2017 ▸). In (I), the inter-centroid separation between *Cg*(C23–C28)⋯*Cg*(Zn,S2,N5,N6,C38)^i^ is 3.5559 (11) Å with an inter-planar angle = 6.70 (8)° and slippage of 0.34 Å for symmetry operation (i): 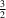 − *x*, 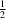 + *y*, 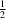 − *z*. The links between chains to consolidate the three-dimensional architecture are of the type phenyl-C14—H⋯O1(nitro) and nitro-O1⋯π(phen­yl), Table 2 ▸. The parameters associated with the latter inter­action are: N4—O1⋯*Cg*(C23–C28)^ii^ = 3.4788 (19) Å with angle at O1 = 108.71 (13)° for (ii): 1 − *x*, 1 − *y*, 1 − *z*. A view of the unit-cell contents is shown in Fig. 3 ▸.

## Analysis of the Hirshfeld surfaces   

**Figure 4:**
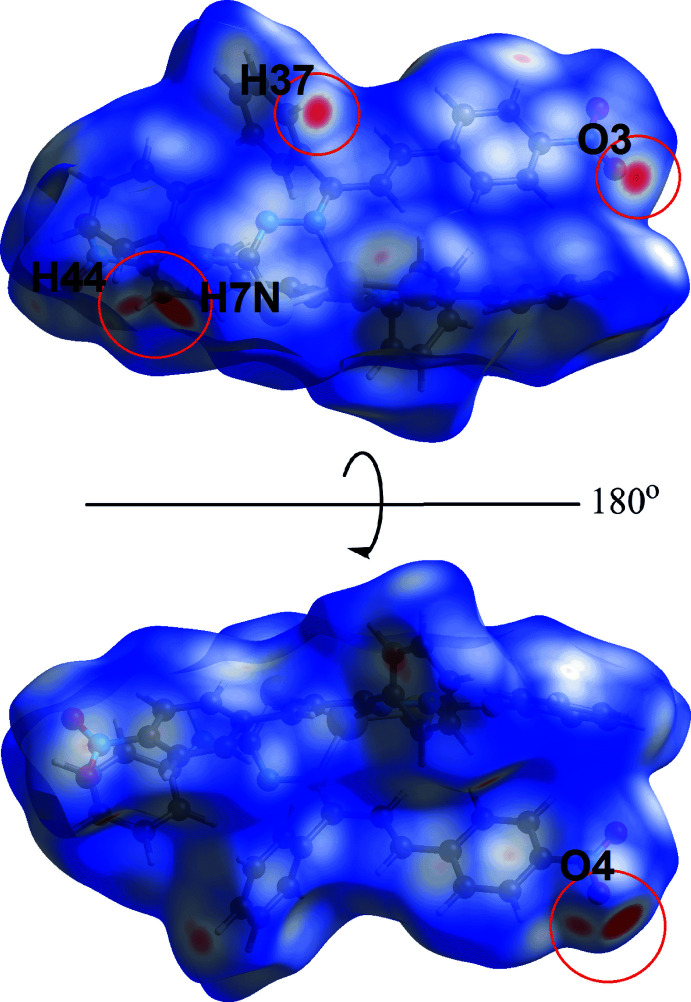
Two views of the Hirshfeld surface mapped over *d*_norm_ for (I) in the range −0.239 to +1.045 arbitrary units, highlighting N—H⋯O and C—H⋯O contact within red circles.

**Figure 5:**
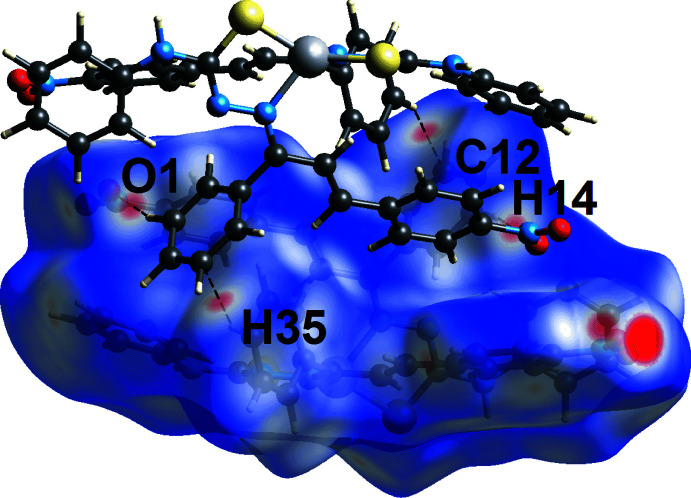
View of the Hirshfeld surface mapped over *d*_norm_ for (I), highlighting inter-chain C—H⋯O and C—H⋯C inter­actions.

In order to acquire further information on the supra­molecular association between mol­ecules in the crystal of (I), the Hirshfeld surface and two-dimensional fingerprint plots were calculated employing the program *Crystal Explorer 17* (Turner *et al.*, 2017 ▸) employing established methods (Tan *et al.*, 2019 ▸). The bright-red spots on the Hirshfeld surface mapped over *d*_norm_ in Fig. 4 ▸, *i.e.* near the amine-H7*N*, phenyl-H44 and nitro-O4 atoms correspond to the inter­actions leading to the linear chain; geometric data for the identified contacts in the Hirshfeld surface analysis are given in Table 3 ▸. Links between chains include phenyl-C37—H⋯O3 (Fig. 4 ▸), phenyl-C14—H⋯O1 and phenyl-C35—H⋯C12 inter­actions (Fig. 5 ▸) and these shown as red spots on the *d*_norm_-mapped Hirshfeld surfaces in Figs. 4 ▸ and 5 ▸.

**Figure 6:**
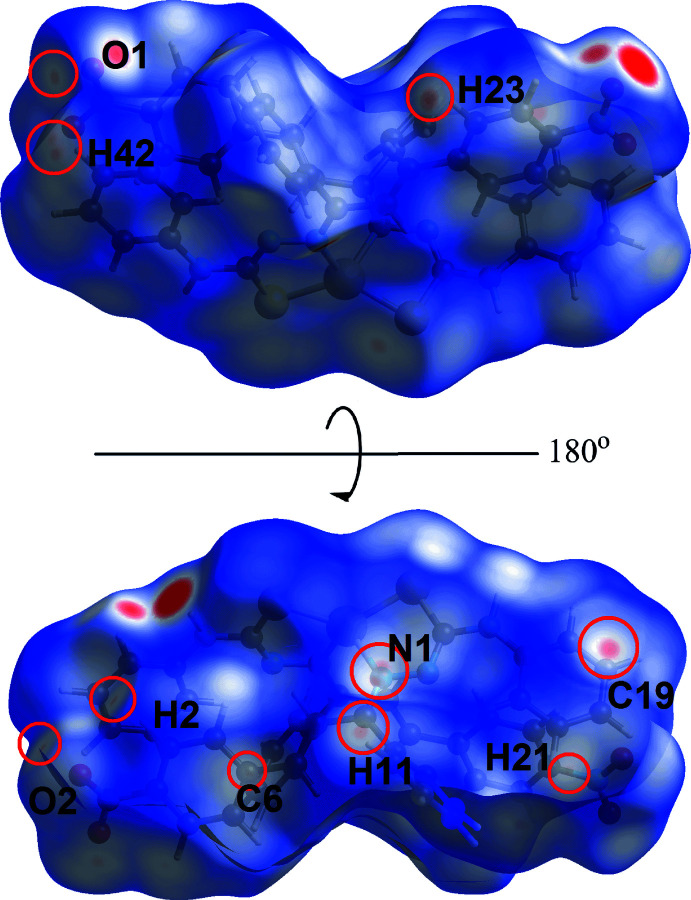
Two views of the Hirshfeld surface mapped over *d*_norm_ for (I), highlighting weak inter­actions within red circles (see text).

**Figure 7:**
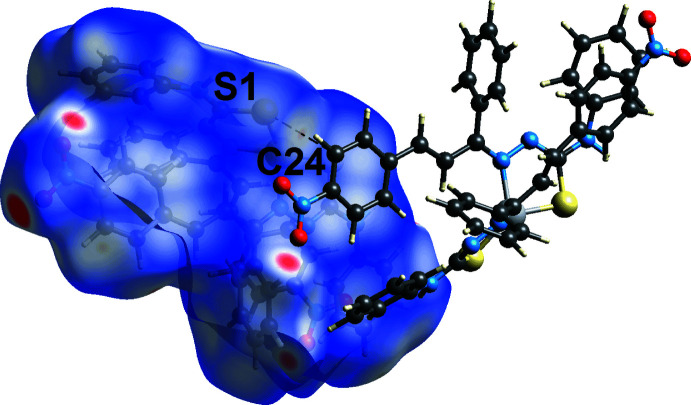
View of the Hirshfeld surface mapped over *d*_norm_ for (I), highlighting C⋯S short contacts.

**Figure 8:**
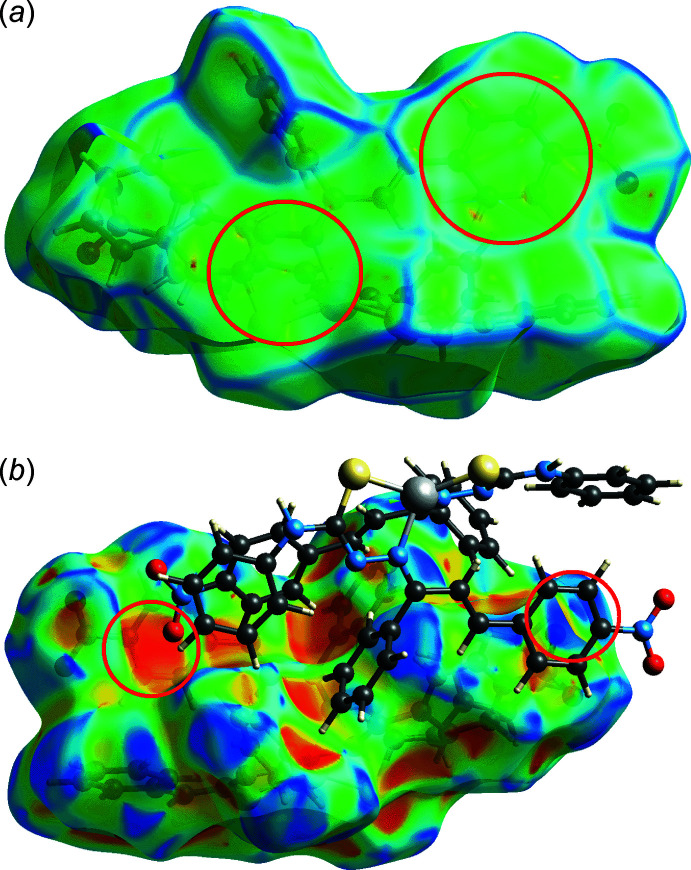
Views of the Hirshfeld surface mapped over (*a*) curveness and (*b*) the shape index property highlighting the inter­molecular π–π and N—O⋯π inter­actions, respectively.

The faint-red spots observed on the *d*_norm_-mapped Hirshfeld surface of Fig. 6 ▸ correspond to a number of weak contacts listed in Table 3 ▸. In addition, an extra C24⋯S1 short contact was observed in the mol­ecular packing, Fig. 7 ▸, with a distance of 0.05 Å shorter than the sum of their van der Waals radii, Table 3 ▸. The π(C23–C28)–π(Zn,S2,N5,N6,C38) and nitro-O1⋯π(C23–C28) inter­actions were not manifested on the *d*_norm_-mapped Hirshfeld surface. However, the π–π inter­action appears as a flat surface on the curvedness-mapped Hirshfeld surface of Fig. 8 ▸(*a*), the nitro-O⋯π inter­action is shown as red concave and blue bump regions on the shape-index-mapped Hirshfeld surface of Fig. 8 ▸(*b*).

**Figure 9:**
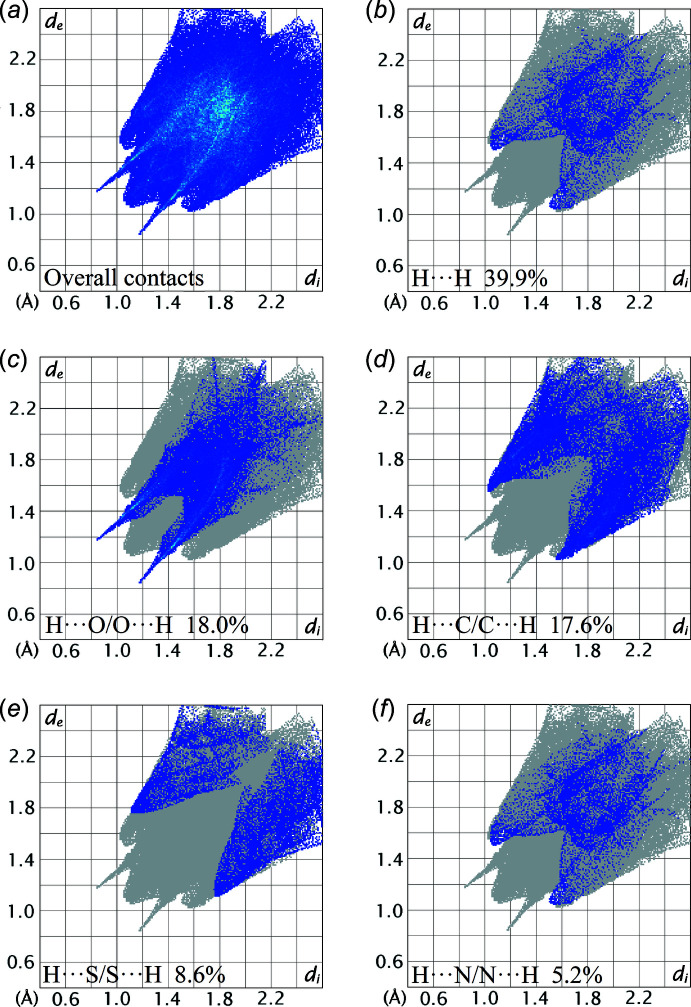
(*a*) A comparison of the full two-dimensional fingerprint plot for (I) and those delineated into (*b*) H⋯H, (*c*) H⋯O/O⋯H, (*d*) H⋯C/C⋯H, (*e*) H⋯S/S⋯H and (*f*) H⋯N/N⋯H contacts.

The overall two-dimensional fingerprint plot for (I) along with those delineated into the individual H⋯H, H⋯O/O⋯H, H⋯C/C⋯H, H⋯S/S⋯H and H⋯N/N⋯H contacts are illustrated in Fig. 9 ▸(*a*)–(*f*), respectively. The percentage contributions from each inter­atomic contact are summarized in Table 4 ▸. As the greatest contributor to the overall Hirshfeld surface, the H⋯H contacts contributed 39.9%, Fig. 9 ▸(*b*), with the peak tipped at *d*_e_ = *d*_i_ ∼2.2 Å corresponding to the H24⋯H44 contact, Table 3 ▸. Consistent with the C—H⋯O and C—H⋯C inter­actions manifested in the mol­ecular packing, H⋯O/O⋯H and H⋯C/C⋯H contacts are the next most prominent, with percentage contributions of 18.0 and 17.6% to the overall surface, with the peak of these contacts tipped at *d*_e_ + *d*_i_ ∼2.0 and 2.6 Å, respectively, Fig. 9 ▸(*c*) and (*d*). The H⋯S/S⋯H contacts contribute 8.6% and appear as two blunt-symmetric wings at *d*_e_ + *d*_i_ ∼2.9 Å in Fig. 9 ▸(*e*). This feature reflects the long-range H⋯S/S⋯H contact evinced in the packing with a separation of 0.1 Å shorter than the sum of their van der Waals radii, Table 3 ▸. Although H⋯N/N⋯H contacts appear at *d*_e_ + *d*_i_ ∼2.6 Å in the fingerprint plot of Fig. 9 ▸(*f*), the contribution to the overall Hirshfeld surface is only 5.2%. The other 11 inter­atomic contacts have a negligible effect on the mol­ecular packing as their accumulated contribution is below 11%, Table 4 ▸.

## Computational chemistry   

**Figure 10:**
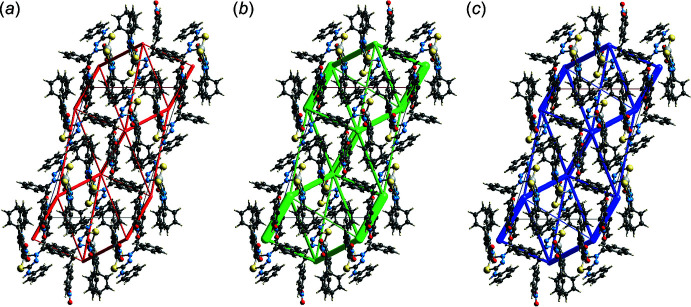
Perspective views of the energy frameworks calculated for (I) showing (*a*) electrostatic potential force, (*b*) dispersion force and (*c*) total energy, each plotted down the *b* axis. The radii of the cylinders are proportional to the relative magnitudes of the corresponding energies and were adjusted to the same scale factor of 50 with a cut-off value of 5 kJ mol^−1^ within 1 × 1 × 1 unit-cells.

The pairwise inter­action energies between mol­ecules in the mol­ecular packing of (I) were calculated using wave-functions at the B3LYP/6-31G(*d*,*p*) level of theory. The total energy (*E*_tot_) was calculated by summing four energy components, comprising the electrostatic (*E*_ele_), polarization (*E*_pol_), dispersion (*E*_dis_) and exchange-repulsion (*E*_rep_) energies. The independent energy components as well as the *E*_tot_ are tabulated in Table 5 ▸. Even with the presence of hydrogen bonds, the *E*_dis_ energy term still makes the major contribution to the inter­action energies partly due to the presence of π–π, N—O⋯π, C—H⋯O and C—H⋯C inter­actions. The total *E*_dis_ components of all pairwise inter­actions sum to −432.1 kJ mol^−1^, whereas the total *E*_ele_ sums to −190.2 kJ mol^−1^. The stabilization of the crystal through the contribution of the dispersion forces is emphasized by the energy framework diagram, Fig. 10 ▸, viewed down the *b* axis.

## Database survey   

The ligand in (I) may be considered a chalcone–thio­semicarbazone hybrid ligand having elements of both chalcone and thio­semicarbazone. There are four related species in the literature, namely an *N*-bound ethyl species with a terminal phenyl ring [(II); Cambridge Structural Database refcode JAXFEW; Tan *et al.*, 2017 ▸], a terminal 4-meth­oxy­benzene ring [(III); QEMXUE; Tan *et al.*, 2018 ▸] as well as two *N*-bound phenyl derivatives with terminal 4-cyano [(IV); QISJUA; Barbosa *et al.*, 2018 ▸] and 4-chloro rings [(V); QISKEL; Barbosa *et al.*, 2018 ▸]; (V) was characterized as a 1:1 THF solvate. In each of (I)–(V), the imine-bound substituent is a phenyl ring. Selected geometric parameters for (I)–(V), calculated employing *PLATON* (Spek, 2020 ▸), are collated in Table 6 ▸. From the data collated, there is an obvious homogeneity in the data to the point of common disparities in the Zn—S and Zn—N bond lengths formed by the two ligands in each complex. The range of tetra­hedral angles are similar as are the dihedral angles formed between the chelate rings. A measurement of the distortion of a four-coordinate donor set from a regular geometry is qu­anti­fied by the value of τ_4_ (Yang *et al.*, 2007 ▸). The value of τ_4_ is 1.00 for an ideal tetra­hedron and 0.00 for perfect square-planar geometry. The range of values for τ_4_ listed in Table 6 ▸ vindicate the assignment of similar coordination geometries for (I)–(V), being distorted from a regular tetra­hedron.

## Synthesis and crystallization   

Analytical grade reagents were used as procured and without further purification. 4-Phenyl-3-thio­semicarbazide (1.6723 g, 10 mmol) and 4-nitro­chalcone (2.5325 g, 10 mmol) were dissolved separately in hot absolute ethanol (50 ml) and mixed while stirring. About five drops of concentrated hydro­chloric acid were added to the mixture and the mixture was heated (348 K) while stirring for about 30 min. The yellow precipitate, (2*E*)-2-[3-(4-nitro­phen­yl)-1-phenyl­allyl­idene]-*N*-phenyl­hydrazine-1-carbo­thio­amide, (VI), was filtered, washed with cold ethanol and dried *in vacuo* after which it was used without further purification. Compound (VI) (0.4047 g, 1 mmol) was dissolved in hot absolute ethanol (50 ml), which was added to a solution of Zn(CH_3_COO)_2_·2H_2_O (0.1098 g, 0.5 mmol) in hot absolute ethanol (40 ml). The mixture was heated (348 K) and stirred for about 10 min, followed by stirring for about 1 h at room temperature. The white precipitate obtained was filtered, washed with cold ethanol and dried *in vacuo*. Single crystals were grown at room temperature by slow evaporation of (I) in a mixed solvent system containing methanol and aceto­nitrile (1:1; *v*/*v* 20 ml). Yield: 90%, m.p. 511–512 K. FT–IR (ATR (solid) cm^−1^): 3428 ν(N—H), 1593 ν(C=N), 1335 ν(N—N), 579 ν(Zn—N), 489 ν(Zn—S). UV–Visible: λ_max_ (nm; ɛ (L mol^−1^ cm^−1^)): 250 (25,070), 292 (13,010), 433 (21,810). ICP–AES: Experimental %Zn = 7.26, Theoretical %Zn = 7.53.

## Refinement   

Crystal data, data collection and structure refinement details are summarized in Table 7 ▸. The carbon-bound H atoms were placed in calculated positions (C—H = 0.95 Å) and were included in the refinement in the riding-model approximation, with *U*_iso_(H) set to 1.2*U*_eq_(C). The *N*-bound H atoms were located in a difference-Fourier map, but were refined with an N—H = 0.88±0.01 Å distance restraint, and with *U*_iso_(H) set to 1.2*U*_eq_(N).

## Supplementary Material

Crystal structure: contains datablock(s) I, global. DOI: 10.1107/S2056989021007398/mw2178sup1.cif

Structure factors: contains datablock(s) I. DOI: 10.1107/S2056989021007398/mw2178Isup2.hkl

CCDC reference: 2097106

Additional supporting information:  crystallographic information; 3D view; checkCIF report

## Figures and Tables

**Table 1:** Selected geometric parameters (Å, °)

Zn—S1	2.2558 (5)	Zn—S2	2.2618 (5)
Zn—N1	2.0757 (16)	Zn—N5	2.0688 (16)
S1—C16	1.758 (2)	S2—C38	1.759 (2)
N1—N2	1.382 (2)	N5—N6	1.381 (2)
N2—C16	1.314 (2)	N6—C38	1.309 (2)
N3—C16	1.360 (2)	N7—C38	1.363 (2)
			
S1—Zn—S2	131.16 (2)	S2—Zn—N1	125.14 (4)
S1—Zn—N1	86.77 (4)	S2—Zn—N5	87.56 (4)
S1—Zn—N5	127.38 (5)	N1—Zn—N5	98.00 (6)

**Table 2:** Hydrogen-bond geometry (Å, °)

*D*—H⋯*A*	*D*—H	H⋯*A*	*D*⋯*A*	*D*—H⋯*A*
N7—H7*N*⋯O4^i^	0.87 (2)	2.18 (2)	3.019 (2)	165 (2)
C44—H44⋯O4^i^	0.95	2.49	3.305 (3)	144
C37—H37⋯O3^ii^	0.95	2.48	3.373 (3)	157
C14—H14⋯O1^iii^	0.95	2.54	3.462 (3)	164

Symmetry codes: (i) x, y-1, z; (ii) -x+{\script{3\over 2}}, y-{\script{1\over 2}}, -z+{\script{1\over 2}}; (iii) -x+1, -y+1, -z+1.

**Table 3:** A summary of short inter­atomic contacts (Å) for (I)*^*a*^*

Contact	Distance	Symmetry operation
N7—H7*N*⋯O4*^*b*^*	2.04	*x*, *y* − 1, *z*
C44—H44⋯O4*^*b*^*	2.38	*x*, *y* − 1, *z*
C37—H37⋯O3*^*b*^*	2.36	−*x* + {3\over 2}, *y* − {1\over 2}, −*z* + {1\over 2}
C14—H14⋯O1*^*b*^*	2.41	−*x* + 1, −*y* + 1, −*z* + 1
C35—H35⋯C12	2.60	−*x* + 1, −*y* + 1, −*z* + 1
C2—H2⋯O2	2.54	*x*, *y*, *z*
H24⋯H44	2.17	*x*, *y* − 1, *z*
S1⋯H24	2.93	−*x* + {3\over 2}, *y* − {1\over 2}, −*z* + {1\over 2}
C23—H23⋯C19	2.66	*x* + {1\over 2}, −*y* + {3\over 2}, *z* + {1\over 2}
C11—H11⋯O1	2.54	*x* − {1\over 2}, −*y* + {1\over 2}, *z* − {1\over 2}
C42—H42⋯N1	2.59	*x* + {1\over 2}, −*y* + {1\over 2}, *z* + {1\over 2}
C21—H21⋯C6	2.75	−*x* + {1\over 2}, *y* + {1\over 2}, −*z* + {1\over 2}
S1⋯C24	3.45	−*x* + {3\over 2}, *y* − {1\over 2}, −*z* + {1\over 2}

Notes: (*a*) The inter­atomic distances are calculated in *Crystal Explorer 17* (Turner *et al.*, 2017 ▸) with the *X*—H bond lengths adjusted to their neutron values; (*b*) these inter­actions correspond to those listed in Table 2 ▸.

**Table 4:** Percentage contributions of inter­atomic contacts to the calculated Hirshfeld surface of (I)

Contact	Percentage contribution	Contact	Percentage contribution
H⋯H	39.9	C⋯N/N⋯C	1.5
H⋯O/O⋯H	18.0	C⋯Zn/Zn⋯C	0.9
H⋯C/C⋯H	17.6	H⋯Zn/Zn⋯H	0.5
H⋯S/S⋯H	8.6	O⋯O	0.4
H⋯N/N⋯H	5.2	O⋯N/N⋯O	0.4
C⋯S/S⋯C	2.4	N⋯N	0.3
C⋯C	1.9	O⋯S/S⋯O	0.3
C⋯O/O⋯C	1.8	N⋯S/S⋯N	0.3

**Table 5:** A summary of inter­action energies (kJ mol^−1^) calculated for (I)

Contact	*R* (Å)	*E*_ele_	*E*_pol_	*E*_dis_	*E*_rep_	*E*_tot_
C14—H14⋯O1^i^ +						
C35—H35⋯C12^i^ +						
N4—O1⋯*Cg*1^i^ +						
H4⋯H15^i^	7.91	−44.4	−12.4	−140.2	146.0	−88.0
C37—H37⋯O3^ii^ +						
*Cg*1⋯*Cg*2^iii^ +						
S1⋯H24^ii^ +						
H19⋯H36^iii^	10.18	−36.9	−5.1	−83.6	78.3	−67.2
C2—H2⋯O2^iv^	16.54	−14.3	−4.0	−20.9	15.4	−26.8
N7—H7*N*⋯O4^v^ +						
C44—H44⋯O4^v^ +						
H24⋯H44^vi^	15.83	−22.5	−5.8	−15.2	32.7	−21.1
C42—H42⋯N1^vii^ +						
C11—H11⋯O1^viii^	12.05	−16.9	−3.3	−43.8	33.0	−38.0
C12—H12⋯O4^ix^ +						
C21—H21⋯C6^*x*^	11.21	−11.4	−3.5	−48.8	37.4	−34.0
C23—H23⋯C19^xi^ +						
H19⋯H29^xii^	13.60	−16.5	−3.4	−32.6	29.0	−30.5
N3—H3*N*⋯S1^xiii^	12.14	−23.3	−3.5	−27.7	35.1	−29.7
H36⋯H37^xiv^	11.81	−4.0	−1.0	−19.3	15.8	−12.0

Symmetry code: (i) −*x* + 1, −*y* + 1, −*z* + 1; (ii) −*x* + {3\over 2}, *y* − {1\over 2}, −*z* + {1\over 2}; (iii) −*x* + {3\over 2}, *y* + {1\over 2}, −*z* + {1\over 2}; (iv) −*x* + 1, −*y*, −*z* + 1; (v) *x*, *y* − 1, *z*; (vi) *x*, *y* + 1, *z*; (vii) *x* + {1\over 2}, −*y* + {1\over 2}, *z* + {1\over 2}; (viii) *x* − {1\over 2}, −*y* + {1\over 2}, *z* − {1\over 2}; (ix) −*x* + {1\over 2}, *y* − {1\over 2}, −*z* + {1\over 2}; (*x*) −*x* + {1\over 2}, *y* + {1\over 2}, −*z* + {1\over 2}; (xi) *x* + {1\over 2}, −*y* + {3\over 2}, *z* + {1\over 2}, (xii) *x* + {1\over 2}, −*y* + {3\over 2}, *z* − {1\over 2}; (xiii) −*x* + 1, −*y* + 1, *z* − {1\over 2}; (xiv) −*x* + 2, −*y* + 1, *z* − {1\over 2}.

**Table 6:** A comparison of key geometric parameters (Å, °) in structures related to (I)

Compound	Zn—S, N (chelate 1)	Zn—S, N (chelate 2)	range of *X*—Zn—*Y* angles	chelate 1/chelate 2 angle	τ_4_	Ref.
(I)	2.2558 (5), 2.0757 (16)	2.2618 (5), 2.0688 (16)	86.77 (4)–131.16 (2)	73.28 (3)	0.72	This work
(II)*^*a*^*	2.2825 (8), 2.0526 (17)	2.2689 (7), 2.0523 (17)	87.00 (5)–133.99 (5)	73.49 (6)	0.70	Tan *et al.* (2017 ▸)
	2.2706 (7), 2.0727 (17)	2.2824 (9), 2.0495 (17)	85.99 (5)–131.30 (6)	77.00 (6)	0.74	
(III)	2.2880 (12), 2.042 (3)	2.2758 (10), 2.070 (3)	86.73 (9)–127.92 (5)	79.68 (13)	0.74	Tan *et al.* (2018 ▸)
(IV)	2.2524 (10), 2.073 (3)	2.2493 (9), 2.060 (2)	87.06 (7)–128.55 (4)	76.11 (9)	0.74	Barbosa *et al.* (2018 ▸)
(V)	2.2636 (7), 2.068 (2)	2.2529 (8), 2.041 (2)	86.41 (6)–128.29 (6)	78.82 (8)	0.73	Barbosa *et al.* (2018 ▸)

Note: (*a*) Two independent mol­ecules comprise the asymmetric unit.

**Table 7:** Experimental details

Crystal data	
Chemical formula	[Zn(C_22_H_17_N_4_O_2_S)_2_]
*M*_r_	868.28
Crystal system, space group	Monoclinic, *P*2_1_/*n*
Temperature (K)	100
*a*, *b*, *c* (Å)	13.4029 (4), 15.8310 (4), 19.6257 (6)
β (°)	107.841 (3)
*V* (Å^3^)	3964.0 (2)
*Z*	4
Radiation type	Mo *K*α
μ (mm^−1^)	0.78
Crystal size (mm)	0.34 × 0.17 × 0.12

Data collection	
Diffractometer	Oxford Diffraction Gemini
Absorption correction	Multi-scan (*CrysAlis PRO*; Agilent, 2012 ▸)
*T*_min_, *T*_max_	0.865, 1.000
No. of measured, independent and observed [*I* > 2σ(*I*)] reflections	17644, 8932, 7199
*R*_int_	0.032
(sin θ/λ)_max_ (Å^−1^)	0.679

Refinement	
*R*[*F* ^2^ > 2σ(*F* ^2^)], *wR*(*F* ^2^), *S*	0.036, 0.086, 1.02
No. of reflections	8932
No. of parameters	540
No. of restraints	2
H-atom treatment	H atoms treated by a mixture of independent and constrained refinement
Δρ_max_, Δρ_min_ (e Å^−3^)	0.36, −0.40

Computer programs: *CrysAlis PRO* (Agilent, 2012 ▸), *SHELXT* (Sheldrick, 2015*a* ▸), *SHELXL2018/3* (Sheldrick, 2015*b* ▸), *ORTEP-3 for Windows* (Farrugia, 2012 ▸), *DIAMOND* (Brandenburg, 2006 ▸) and *publCIF* (Westrip, 2010 ▸).

## supplementary crystallographic information

### Crystal data

**Table:**

[Zn(C_22_H_17_N_4_O_2_S)_2_]	*F*(000) = 1792
*M**_r_* = 868.28	*D*_x_ = 1.455 Mg m^−^^3^
Monoclinic, *P*2_1_/*n*	Mo *K*α radiation, λ = 0.71073 Å
*a* = 13.4029 (4) Å	Cell parameters from 5987 reflections
*b* = 15.8310 (4) Å	θ = 2.2–28.8°
*c* = 19.6257 (6) Å	µ = 0.78 mm^−^^1^
β = 107.841 (3)°	*T* = 100 K
*V* = 3964.0 (2) Å^3^	Prism, colourless
*Z* = 4	0.34 × 0.17 × 0.12 mm

### Data collection

**Table:**

Oxford Diffraction Gemini diffractometer	7199 reflections with *I* > 2σ(*I*)
Graphite monochromator	*R*_int_ = 0.032
ω scans	θ_max_ = 28.9°, θ_min_ = 2.2°
Absorption correction: multi-scan (CrysAlisPro; Agilent, 2012)	*h* = −16→17
*T*_min_ = 0.865, *T*_max_ = 1.000	*k* = −17→19
17644 measured reflections	*l* = −26→23
8932 independent reflections	

### Refinement

**Table:**

Refinement on *F*^2^	Primary atom site location: structure-invariant direct methods
Least-squares matrix: full	Secondary atom site location: difference Fourier map
*R*[*F*^2^ > 2σ(*F*^2^)] = 0.036	Hydrogen site location: mixed
*wR*(*F*^2^) = 0.086	H atoms treated by a mixture of independent and constrained refinement
*S* = 1.01	*w* = 1/[σ^2^(*F*_o_^2^) + (0.0322*P*)^2^ + 1.7875*P*] where *P* = (*F*_o_^2^ + 2*F*_c_^2^)/3
8932 reflections	(Δ/σ)_max_ = 0.001
540 parameters	Δρ_max_ = 0.36 e Å^−^^3^
2 restraints	Δρ_min_ = −0.40 e Å^−^^3^

### Special details

**Table:**

Geometry. All esds (except the esd in the dihedral angle between two l.s. planes) are estimated using the full covariance matrix. The cell esds are taken into account individually in the estimation of esds in distances, angles and torsion angles; correlations between esds in cell parameters are only used when they are defined by crystal symmetry. An approximate (isotropic) treatment of cell esds is used for estimating esds involving l.s. planes.

### Fractional atomic coordinates and isotropic or equivalent isotropic displacement parameters (Å^2^)

**Table:**

	*x*	*y*	*z*	*U*_iso_*/*U*_eq_	
Zn	0.58274 (2)	0.37086 (2)	0.20624 (2)	0.01673 (7)	
S1	0.58652 (4)	0.44525 (3)	0.10903 (3)	0.02441 (12)	
S2	0.58396 (4)	0.23003 (3)	0.22591 (3)	0.01931 (11)	
O1	0.55672 (12)	0.15118 (10)	0.65080 (8)	0.0303 (4)	
O2	0.50717 (14)	0.05390 (9)	0.57014 (9)	0.0369 (4)	
O3	0.54146 (13)	0.94898 (9)	0.17974 (8)	0.0329 (4)	
O4	0.61311 (15)	0.99625 (9)	0.28598 (9)	0.0420 (4)	
N1	0.46644 (12)	0.45354 (9)	0.21386 (8)	0.0158 (3)	
N2	0.44712 (12)	0.52228 (10)	0.16827 (8)	0.0168 (3)	
N3	0.48578 (14)	0.58845 (11)	0.07532 (9)	0.0214 (4)	
H3N	0.5220 (15)	0.5835 (13)	0.0455 (10)	0.020 (6)*	
N4	0.52155 (14)	0.12782 (11)	0.58837 (10)	0.0238 (4)	
N5	0.68182 (12)	0.38332 (9)	0.30981 (8)	0.0154 (3)	
N6	0.70509 (13)	0.30949 (9)	0.34901 (9)	0.0171 (3)	
N7	0.68388 (14)	0.16658 (10)	0.35245 (9)	0.0199 (4)	
H7N	0.6565 (16)	0.1232 (10)	0.3266 (10)	0.026 (6)*	
N8	0.59028 (13)	0.93859 (10)	0.24236 (9)	0.0212 (4)	
C1	0.45854 (15)	0.23217 (12)	0.41034 (11)	0.0202 (4)	
H1	0.453813	0.217926	0.362427	0.024*	
C2	0.48297 (16)	0.17003 (13)	0.46245 (11)	0.0218 (4)	
H2	0.491912	0.112986	0.450476	0.026*	
C3	0.49404 (15)	0.19296 (12)	0.53227 (11)	0.0198 (4)	
C4	0.47873 (15)	0.27476 (12)	0.55129 (11)	0.0202 (4)	
H4	0.487903	0.289001	0.599854	0.024*	
C5	0.44979 (15)	0.33557 (12)	0.49829 (11)	0.0201 (4)	
H5	0.435907	0.391535	0.510350	0.024*	
C6	0.44065 (14)	0.31582 (12)	0.42709 (10)	0.0176 (4)	
C7	0.41199 (15)	0.38247 (12)	0.37269 (10)	0.0179 (4)	
H7	0.373314	0.429169	0.381690	0.022*	
C8	0.43599 (15)	0.38270 (12)	0.31141 (10)	0.0174 (4)	
H8	0.468932	0.333854	0.300077	0.021*	
C9	0.41514 (15)	0.45260 (12)	0.26090 (10)	0.0165 (4)	
C10	0.33978 (15)	0.52098 (12)	0.26386 (10)	0.0168 (4)	
C11	0.23278 (16)	0.50424 (13)	0.24251 (11)	0.0229 (4)	
H11	0.207955	0.448858	0.228010	0.027*	
C12	0.16233 (17)	0.56849 (15)	0.24240 (12)	0.0282 (5)	
H12	0.089173	0.557323	0.226578	0.034*	
C13	0.19822 (18)	0.64882 (14)	0.26526 (11)	0.0281 (5)	
H13	0.149830	0.692385	0.266047	0.034*	
C14	0.30442 (18)	0.66550 (13)	0.28691 (12)	0.0269 (5)	
H14	0.328964	0.720644	0.302532	0.032*	
C15	0.37555 (16)	0.60205 (13)	0.28596 (11)	0.0223 (4)	
H15	0.448522	0.613961	0.300379	0.027*	
C16	0.49833 (15)	0.52179 (12)	0.12093 (10)	0.0181 (4)	
C17	0.43201 (16)	0.66552 (12)	0.07269 (11)	0.0207 (4)	
C18	0.45367 (17)	0.72857 (13)	0.02964 (11)	0.0257 (5)	
H18	0.499997	0.717405	0.002519	0.031*	
C19	0.40772 (17)	0.80745 (14)	0.02638 (12)	0.0304 (5)	
H19	0.422299	0.849898	−0.003447	0.037*	
C20	0.34085 (18)	0.82520 (14)	0.06602 (13)	0.0332 (5)	
H20	0.310569	0.879725	0.064307	0.040*	
C21	0.31882 (17)	0.76236 (14)	0.10812 (13)	0.0315 (5)	
H21	0.272830	0.774161	0.135360	0.038*	
C22	0.36257 (16)	0.68213 (13)	0.11149 (12)	0.0259 (5)	
H22	0.345390	0.639214	0.139829	0.031*	
C23	0.70309 (15)	0.76045 (12)	0.36476 (11)	0.0194 (4)	
H23	0.740369	0.751645	0.413851	0.023*	
C24	0.67607 (15)	0.84148 (12)	0.33996 (11)	0.0195 (4)	
H24	0.694471	0.888503	0.371405	0.023*	
C25	0.62165 (15)	0.85267 (11)	0.26834 (11)	0.0169 (4)	
C26	0.59400 (15)	0.78618 (12)	0.22071 (11)	0.0195 (4)	
H26	0.557032	0.795631	0.171657	0.023*	
C27	0.62175 (16)	0.70522 (12)	0.24655 (11)	0.0203 (4)	
H27	0.603312	0.658577	0.214698	0.024*	
C28	0.67624 (14)	0.69108 (11)	0.31847 (10)	0.0160 (4)	
C29	0.70270 (15)	0.60651 (12)	0.34873 (11)	0.0182 (4)	
H29	0.733633	0.603231	0.399152	0.022*	
C30	0.68795 (15)	0.53321 (12)	0.31287 (11)	0.0174 (4)	
H30	0.662728	0.534603	0.262062	0.021*	
C31	0.70882 (14)	0.45193 (11)	0.34831 (10)	0.0161 (4)	
C32	0.75469 (15)	0.44706 (11)	0.42785 (10)	0.0172 (4)	
C33	0.68844 (17)	0.44340 (12)	0.47036 (11)	0.0232 (4)	
H33	0.614641	0.445966	0.448801	0.028*	
C34	0.72993 (18)	0.43605 (13)	0.54392 (12)	0.0276 (5)	
H34	0.684477	0.432751	0.572662	0.033*	
C35	0.83700 (19)	0.43348 (13)	0.57565 (12)	0.0286 (5)	
H35	0.865457	0.429202	0.626204	0.034*	
C36	0.90223 (18)	0.43714 (14)	0.53374 (12)	0.0306 (5)	
H36	0.975996	0.435308	0.555597	0.037*	
C37	0.86174 (17)	0.44350 (14)	0.45986 (11)	0.0259 (5)	
H37	0.907621	0.445393	0.431367	0.031*	
C38	0.66455 (15)	0.24045 (12)	0.31503 (10)	0.0166 (4)	
C39	0.72045 (15)	0.15319 (12)	0.42737 (11)	0.0198 (4)	
C40	0.76208 (17)	0.21521 (14)	0.47840 (11)	0.0277 (5)	
H40	0.773700	0.270666	0.463945	0.033*	
C41	0.78648 (18)	0.19551 (14)	0.55048 (12)	0.0314 (5)	
H41	0.813842	0.238257	0.585148	0.038*	
C42	0.77190 (18)	0.11516 (14)	0.57297 (12)	0.0306 (5)	
H42	0.787784	0.102876	0.622539	0.037*	
C43	0.73386 (19)	0.05284 (14)	0.52240 (13)	0.0334 (5)	
H43	0.725650	−0.003121	0.537320	0.040*	
C44	0.70773 (18)	0.07131 (13)	0.45040 (12)	0.0286 (5)	
H44	0.680855	0.028083	0.416101	0.034*	

### Atomic displacement parameters (Å^2^)

**Table:**

	*U*^11^	*U*^22^	*U*^33^	*U*^12^	*U*^13^	*U*^23^
Zn	0.02270 (13)	0.01347 (11)	0.01473 (12)	0.00168 (9)	0.00678 (9)	0.00058 (9)
S1	0.0346 (3)	0.0236 (3)	0.0196 (3)	0.0088 (2)	0.0151 (2)	0.0058 (2)
S2	0.0269 (3)	0.0131 (2)	0.0173 (2)	−0.00068 (19)	0.0060 (2)	−0.00247 (19)
O1	0.0369 (9)	0.0309 (9)	0.0207 (8)	0.0035 (7)	0.0053 (7)	0.0061 (7)
O2	0.0563 (11)	0.0170 (8)	0.0372 (10)	−0.0021 (7)	0.0139 (8)	0.0060 (7)
O3	0.0496 (10)	0.0212 (8)	0.0222 (8)	0.0083 (7)	0.0025 (7)	0.0060 (6)
O4	0.0681 (12)	0.0114 (8)	0.0320 (9)	0.0003 (7)	−0.0060 (8)	−0.0031 (7)
N1	0.0194 (8)	0.0127 (8)	0.0139 (8)	−0.0009 (6)	0.0030 (6)	0.0004 (6)
N2	0.0207 (8)	0.0145 (8)	0.0143 (8)	0.0006 (6)	0.0037 (7)	0.0025 (6)
N3	0.0299 (10)	0.0202 (9)	0.0152 (9)	0.0025 (7)	0.0088 (7)	0.0040 (7)
N4	0.0250 (9)	0.0220 (9)	0.0259 (10)	0.0017 (7)	0.0100 (8)	0.0075 (8)
N5	0.0169 (8)	0.0125 (8)	0.0172 (8)	0.0014 (6)	0.0057 (7)	0.0018 (6)
N6	0.0221 (8)	0.0109 (8)	0.0180 (8)	0.0017 (6)	0.0057 (7)	0.0016 (6)
N7	0.0288 (10)	0.0101 (8)	0.0196 (9)	−0.0008 (7)	0.0059 (7)	−0.0004 (7)
N8	0.0260 (9)	0.0147 (8)	0.0218 (9)	0.0003 (7)	0.0058 (7)	0.0028 (7)
C1	0.0248 (11)	0.0186 (10)	0.0186 (10)	−0.0005 (8)	0.0089 (8)	−0.0013 (8)
C2	0.0247 (11)	0.0168 (10)	0.0245 (11)	0.0002 (8)	0.0085 (9)	−0.0006 (8)
C3	0.0184 (10)	0.0194 (10)	0.0217 (11)	0.0006 (8)	0.0064 (8)	0.0060 (8)
C4	0.0228 (10)	0.0222 (10)	0.0160 (10)	−0.0010 (8)	0.0066 (8)	0.0005 (8)
C5	0.0224 (10)	0.0172 (10)	0.0219 (11)	0.0000 (8)	0.0088 (8)	−0.0012 (8)
C6	0.0149 (9)	0.0204 (10)	0.0182 (10)	−0.0026 (8)	0.0061 (8)	0.0015 (8)
C7	0.0183 (10)	0.0154 (9)	0.0202 (10)	0.0001 (7)	0.0060 (8)	0.0001 (8)
C8	0.0178 (9)	0.0145 (9)	0.0202 (10)	−0.0003 (7)	0.0060 (8)	0.0000 (8)
C9	0.0174 (10)	0.0150 (9)	0.0154 (9)	−0.0023 (7)	0.0027 (8)	−0.0026 (8)
C10	0.0193 (10)	0.0186 (10)	0.0129 (9)	0.0013 (8)	0.0053 (8)	0.0011 (8)
C11	0.0221 (11)	0.0244 (11)	0.0211 (11)	−0.0004 (8)	0.0051 (9)	0.0000 (9)
C12	0.0200 (11)	0.0385 (13)	0.0244 (11)	0.0047 (9)	0.0045 (9)	0.0031 (10)
C13	0.0331 (12)	0.0309 (12)	0.0214 (11)	0.0153 (10)	0.0098 (10)	0.0063 (9)
C14	0.0366 (13)	0.0192 (10)	0.0253 (12)	0.0031 (9)	0.0103 (10)	−0.0005 (9)
C15	0.0233 (10)	0.0204 (10)	0.0240 (11)	−0.0005 (8)	0.0083 (9)	−0.0019 (9)
C16	0.0221 (10)	0.0157 (9)	0.0149 (10)	−0.0015 (8)	0.0032 (8)	0.0002 (8)
C17	0.0216 (10)	0.0173 (10)	0.0181 (10)	0.0006 (8)	−0.0016 (8)	0.0036 (8)
C18	0.0291 (12)	0.0218 (11)	0.0208 (11)	−0.0046 (9)	−0.0002 (9)	0.0043 (9)
C19	0.0287 (12)	0.0222 (11)	0.0303 (12)	−0.0058 (9)	−0.0059 (10)	0.0088 (9)
C20	0.0258 (12)	0.0200 (11)	0.0448 (15)	0.0033 (9)	−0.0024 (10)	0.0052 (10)
C21	0.0228 (11)	0.0278 (12)	0.0415 (14)	0.0060 (9)	0.0062 (10)	0.0071 (10)
C22	0.0212 (11)	0.0220 (11)	0.0317 (12)	0.0026 (8)	0.0039 (9)	0.0079 (9)
C23	0.0210 (10)	0.0166 (10)	0.0181 (10)	0.0002 (8)	0.0023 (8)	−0.0001 (8)
C24	0.0225 (10)	0.0128 (9)	0.0222 (11)	−0.0016 (8)	0.0055 (8)	−0.0028 (8)
C25	0.0189 (10)	0.0113 (9)	0.0213 (10)	0.0003 (7)	0.0071 (8)	0.0023 (8)
C26	0.0231 (10)	0.0169 (10)	0.0174 (10)	−0.0016 (8)	0.0046 (8)	0.0005 (8)
C27	0.0242 (11)	0.0141 (9)	0.0219 (11)	−0.0021 (8)	0.0061 (9)	−0.0026 (8)
C28	0.0149 (9)	0.0133 (9)	0.0199 (10)	−0.0015 (7)	0.0056 (8)	0.0009 (8)
C29	0.0189 (10)	0.0170 (9)	0.0179 (10)	−0.0008 (8)	0.0044 (8)	0.0012 (8)
C30	0.0186 (10)	0.0150 (9)	0.0185 (10)	−0.0017 (8)	0.0056 (8)	0.0004 (8)
C31	0.0153 (9)	0.0137 (9)	0.0198 (10)	0.0011 (7)	0.0061 (8)	0.0010 (8)
C32	0.0218 (10)	0.0098 (9)	0.0189 (10)	−0.0021 (7)	0.0048 (8)	−0.0029 (7)
C33	0.0259 (11)	0.0191 (10)	0.0263 (11)	−0.0031 (8)	0.0107 (9)	−0.0031 (9)
C34	0.0400 (13)	0.0224 (11)	0.0263 (12)	−0.0067 (9)	0.0190 (10)	−0.0047 (9)
C35	0.0448 (14)	0.0220 (11)	0.0171 (11)	−0.0068 (10)	0.0065 (10)	−0.0046 (9)
C36	0.0278 (12)	0.0365 (13)	0.0233 (12)	−0.0033 (10)	0.0018 (9)	−0.0050 (10)
C37	0.0250 (11)	0.0311 (12)	0.0225 (11)	−0.0018 (9)	0.0086 (9)	−0.0022 (9)
C38	0.0207 (10)	0.0133 (9)	0.0168 (10)	0.0022 (7)	0.0071 (8)	0.0003 (8)
C39	0.0205 (10)	0.0178 (10)	0.0202 (10)	0.0052 (8)	0.0052 (8)	0.0047 (8)
C40	0.0329 (12)	0.0236 (11)	0.0215 (11)	−0.0015 (9)	0.0007 (9)	0.0054 (9)
C41	0.0366 (13)	0.0284 (12)	0.0225 (12)	−0.0006 (10)	−0.0006 (10)	0.0011 (10)
C42	0.0306 (12)	0.0360 (13)	0.0221 (11)	0.0091 (10)	0.0038 (9)	0.0118 (10)
C43	0.0463 (15)	0.0221 (11)	0.0310 (13)	0.0076 (10)	0.0107 (11)	0.0125 (10)
C44	0.0412 (13)	0.0164 (10)	0.0282 (12)	0.0032 (9)	0.0108 (10)	0.0020 (9)

### Geometric parameters (Å, º)

**Table:**

Zn—S1	2.2558 (5)	C15—H15	0.9500
Zn—N1	2.0757 (16)	C17—C18	1.395 (3)
S1—C16	1.758 (2)	C17—C22	1.396 (3)
N1—N2	1.382 (2)	C18—C19	1.385 (3)
N2—C16	1.314 (2)	C18—H18	0.9500
N3—C16	1.360 (2)	C19—C20	1.384 (3)
Zn—S2	2.2618 (5)	C19—H19	0.9500
Zn—N5	2.0688 (16)	C20—C21	1.382 (3)
S2—C38	1.759 (2)	C20—H20	0.9500
N5—N6	1.381 (2)	C21—C22	1.392 (3)
N6—C38	1.309 (2)	C21—H21	0.9500
N7—C38	1.363 (2)	C22—H22	0.9500
O1—N4	1.227 (2)	C23—C24	1.380 (3)
O2—N4	1.222 (2)	C23—C28	1.400 (3)
O3—N8	1.214 (2)	C23—H23	0.9500
O4—N8	1.224 (2)	C24—C25	1.382 (3)
N1—C9	1.309 (2)	C24—H24	0.9500
N3—C17	1.410 (3)	C25—C26	1.381 (3)
N3—H3N	0.871 (9)	C26—C27	1.387 (3)
N4—C3	1.471 (2)	C26—H26	0.9500
N5—C31	1.309 (2)	C27—C28	1.394 (3)
N7—C39	1.416 (3)	C27—H27	0.9500
N7—H7N	0.866 (9)	C28—C29	1.464 (3)
N8—C25	1.468 (2)	C29—C30	1.340 (3)
C1—C2	1.384 (3)	C29—H29	0.9500
C1—C6	1.403 (3)	C30—C31	1.449 (3)
C1—H1	0.9500	C30—H30	0.9500
C2—C3	1.381 (3)	C31—C32	1.494 (3)
C2—H2	0.9500	C32—C37	1.380 (3)
C3—C4	1.380 (3)	C32—C33	1.394 (3)
C4—C5	1.383 (3)	C33—C34	1.384 (3)
C4—H4	0.9500	C33—H33	0.9500
C5—C6	1.400 (3)	C34—C35	1.379 (3)
C5—H5	0.9500	C34—H34	0.9500
C6—C7	1.466 (3)	C35—C36	1.373 (3)
C7—C8	1.337 (3)	C35—H35	0.9500
C7—H7	0.9500	C36—C37	1.387 (3)
C8—C9	1.455 (3)	C36—H36	0.9500
C8—H8	0.9500	C37—H37	0.9500
C9—C10	1.494 (3)	C39—C40	1.391 (3)
C10—C11	1.391 (3)	C39—C44	1.400 (3)
C10—C15	1.392 (3)	C40—C41	1.386 (3)
C11—C12	1.387 (3)	C40—H40	0.9500
C11—H11	0.9500	C41—C42	1.380 (3)
C12—C13	1.385 (3)	C41—H41	0.9500
C12—H12	0.9500	C42—C43	1.382 (3)
C13—C14	1.380 (3)	C42—H42	0.9500
C13—H13	0.9500	C43—C44	1.379 (3)
C14—C15	1.389 (3)	C43—H43	0.9500
C14—H14	0.9500	C44—H44	0.9500
			
S1—Zn—S2	131.16 (2)	C19—C18—H18	120.0
S1—Zn—N1	86.77 (4)	C17—C18—H18	120.0
S1—Zn—N5	127.38 (5)	C20—C19—C18	120.8 (2)
S2—Zn—N1	125.14 (4)	C20—C19—H19	119.6
S2—Zn—N5	87.56 (4)	C18—C19—H19	119.6
N1—Zn—N5	98.00 (6)	C21—C20—C19	118.9 (2)
C16—S1—Zn	93.29 (6)	C21—C20—H20	120.5
C38—S2—Zn	92.72 (6)	C19—C20—H20	120.5
C9—N1—N2	115.51 (15)	C20—C21—C22	121.4 (2)
C9—N1—Zn	127.72 (13)	C20—C21—H21	119.3
N2—N1—Zn	116.48 (11)	C22—C21—H21	119.3
C16—N2—N1	114.74 (15)	C21—C22—C17	119.2 (2)
C16—N3—C17	130.84 (17)	C21—C22—H22	120.4
C16—N3—H3N	113.0 (14)	C17—C22—H22	120.4
C17—N3—H3N	115.9 (14)	C24—C23—C28	120.84 (18)
O2—N4—O1	123.98 (18)	C24—C23—H23	119.6
O2—N4—C3	118.13 (18)	C28—C23—H23	119.6
O1—N4—C3	117.89 (17)	C23—C24—C25	118.51 (18)
C31—N5—N6	113.90 (16)	C23—C24—H24	120.7
C31—N5—Zn	128.82 (13)	C25—C24—H24	120.7
N6—N5—Zn	115.72 (11)	C26—C25—C24	122.63 (17)
C38—N6—N5	115.80 (16)	C26—C25—N8	118.83 (17)
C38—N7—C39	129.51 (17)	C24—C25—N8	118.53 (17)
C38—N7—H7N	112.8 (15)	C25—C26—C27	118.09 (18)
C39—N7—H7N	116.1 (15)	C25—C26—H26	121.0
O3—N8—O4	123.27 (17)	C27—C26—H26	121.0
O3—N8—C25	119.05 (16)	C26—C27—C28	121.15 (18)
O4—N8—C25	117.66 (17)	C26—C27—H27	119.4
C2—C1—C6	121.10 (18)	C28—C27—H27	119.4
C2—C1—H1	119.5	C27—C28—C23	118.79 (17)
C6—C1—H1	119.5	C27—C28—C29	122.97 (17)
C3—C2—C1	118.40 (18)	C23—C28—C29	118.17 (17)
C3—C2—H2	120.8	C30—C29—C28	126.92 (18)
C1—C2—H2	120.8	C30—C29—H29	116.5
C4—C3—C2	122.36 (18)	C28—C29—H29	116.5
C4—C3—N4	118.64 (18)	C29—C30—C31	122.77 (18)
C2—C3—N4	118.99 (18)	C29—C30—H30	118.6
C3—C4—C5	118.71 (18)	C31—C30—H30	118.6
C3—C4—H4	120.6	N5—C31—C30	118.76 (17)
C5—C4—H4	120.6	N5—C31—C32	120.84 (16)
C4—C5—C6	120.92 (18)	C30—C31—C32	120.31 (16)
C4—C5—H5	119.5	C37—C32—C33	119.35 (19)
C6—C5—H5	119.5	C37—C32—C31	121.02 (17)
C5—C6—C1	118.41 (18)	C33—C32—C31	119.60 (18)
C5—C6—C7	119.30 (18)	C34—C33—C32	120.1 (2)
C1—C6—C7	122.29 (18)	C34—C33—H33	119.9
C8—C7—C6	125.14 (18)	C32—C33—H33	119.9
C8—C7—H7	117.4	C35—C34—C33	120.2 (2)
C6—C7—H7	117.4	C35—C34—H34	119.9
C7—C8—C9	124.49 (18)	C33—C34—H34	119.9
C7—C8—H8	117.8	C36—C35—C34	119.6 (2)
C9—C8—H8	117.8	C36—C35—H35	120.2
N1—C9—C8	117.16 (17)	C34—C35—H35	120.2
N1—C9—C10	121.78 (17)	C35—C36—C37	120.8 (2)
C8—C9—C10	121.05 (16)	C35—C36—H36	119.6
C11—C10—C15	119.64 (18)	C37—C36—H36	119.6
C11—C10—C9	119.83 (17)	C32—C37—C36	119.9 (2)
C15—C10—C9	120.51 (17)	C32—C37—H37	120.1
C12—C11—C10	120.0 (2)	C36—C37—H37	120.1
C12—C11—H11	120.0	N6—C38—N7	117.47 (17)
C10—C11—H11	120.0	N6—C38—S2	128.14 (15)
C13—C12—C11	120.3 (2)	N7—C38—S2	114.38 (14)
C13—C12—H12	119.9	C40—C39—C44	118.86 (19)
C11—C12—H12	119.9	C40—C39—N7	125.21 (18)
C14—C13—C12	119.9 (2)	C44—C39—N7	115.85 (18)
C14—C13—H13	120.1	C41—C40—C39	119.5 (2)
C12—C13—H13	120.1	C41—C40—H40	120.2
C13—C14—C15	120.3 (2)	C39—C40—H40	120.2
C13—C14—H14	119.8	C42—C41—C40	121.4 (2)
C15—C14—H14	119.8	C42—C41—H41	119.3
C14—C15—C10	119.9 (2)	C40—C41—H41	119.3
C14—C15—H15	120.0	C41—C42—C43	119.1 (2)
C10—C15—H15	120.0	C41—C42—H42	120.5
N2—C16—N3	118.35 (17)	C43—C42—H42	120.5
N2—C16—S1	128.06 (15)	C44—C43—C42	120.4 (2)
N3—C16—S1	113.57 (14)	C44—C43—H43	119.8
C18—C17—C22	119.54 (19)	C42—C43—H43	119.8
C18—C17—N3	116.26 (18)	C43—C44—C39	120.6 (2)
C22—C17—N3	124.18 (18)	C43—C44—H44	119.7
C19—C18—C17	120.1 (2)	C39—C44—H44	119.7
			
C9—N1—N2—C16	−179.30 (17)	C18—C17—C22—C21	−2.0 (3)
Zn—N1—N2—C16	6.4 (2)	N3—C17—C22—C21	176.1 (2)
C31—N5—N6—C38	169.32 (16)	C28—C23—C24—C25	0.1 (3)
Zn—N5—N6—C38	2.3 (2)	C23—C24—C25—C26	−0.5 (3)
C6—C1—C2—C3	−2.9 (3)	C23—C24—C25—N8	178.29 (17)
C1—C2—C3—C4	1.9 (3)	O3—N8—C25—C26	−0.4 (3)
C1—C2—C3—N4	−179.09 (17)	O4—N8—C25—C26	177.99 (19)
O2—N4—C3—C4	160.31 (19)	O3—N8—C25—C24	−179.19 (18)
O1—N4—C3—C4	−19.0 (3)	O4—N8—C25—C24	−0.8 (3)
O2—N4—C3—C2	−18.8 (3)	C24—C25—C26—C27	0.5 (3)
O1—N4—C3—C2	161.88 (18)	N8—C25—C26—C27	−178.27 (17)
C2—C3—C4—C5	1.0 (3)	C25—C26—C27—C28	−0.2 (3)
N4—C3—C4—C5	−178.09 (17)	C26—C27—C28—C23	−0.1 (3)
C3—C4—C5—C6	−2.8 (3)	C26—C27—C28—C29	176.99 (18)
C4—C5—C6—C1	1.9 (3)	C24—C23—C28—C27	0.2 (3)
C4—C5—C6—C7	−178.68 (18)	C24—C23—C28—C29	−177.12 (17)
C2—C1—C6—C5	1.1 (3)	C27—C28—C29—C30	6.2 (3)
C2—C1—C6—C7	−178.38 (18)	C23—C28—C29—C30	−176.68 (19)
C5—C6—C7—C8	154.6 (2)	C28—C29—C30—C31	−174.73 (18)
C1—C6—C7—C8	−26.0 (3)	N6—N5—C31—C30	−179.84 (15)
C6—C7—C8—C9	−174.32 (18)	Zn—N5—C31—C30	−14.9 (2)
N2—N1—C9—C8	−178.00 (15)	N6—N5—C31—C32	−3.3 (2)
Zn—N1—C9—C8	−4.4 (2)	Zn—N5—C31—C32	161.66 (13)
N2—N1—C9—C10	0.9 (3)	C29—C30—C31—N5	172.80 (18)
Zn—N1—C9—C10	174.45 (13)	C29—C30—C31—C32	−3.7 (3)
C7—C8—C9—N1	162.41 (19)	N5—C31—C32—C37	92.3 (2)
C7—C8—C9—C10	−16.5 (3)	C30—C31—C32—C37	−91.2 (2)
N1—C9—C10—C11	109.3 (2)	N5—C31—C32—C33	−85.7 (2)
C8—C9—C10—C11	−71.9 (2)	C30—C31—C32—C33	90.8 (2)
N1—C9—C10—C15	−68.9 (3)	C37—C32—C33—C34	−0.2 (3)
C8—C9—C10—C15	109.9 (2)	C31—C32—C33—C34	177.85 (18)
C15—C10—C11—C12	0.9 (3)	C32—C33—C34—C35	0.9 (3)
C9—C10—C11—C12	−177.35 (18)	C33—C34—C35—C36	−0.9 (3)
C10—C11—C12—C13	−1.7 (3)	C34—C35—C36—C37	0.1 (3)
C11—C12—C13—C14	1.3 (3)	C33—C32—C37—C36	−0.6 (3)
C12—C13—C14—C15	−0.1 (3)	C31—C32—C37—C36	−178.59 (19)
C13—C14—C15—C10	−0.7 (3)	C35—C36—C37—C32	0.7 (3)
C11—C10—C15—C14	0.3 (3)	N5—N6—C38—N7	−178.58 (15)
C9—C10—C15—C14	178.54 (18)	N5—N6—C38—S2	−0.2 (3)
N1—N2—C16—N3	−178.24 (16)	C39—N7—C38—N6	16.9 (3)
N1—N2—C16—S1	0.2 (3)	C39—N7—C38—S2	−161.73 (16)
C17—N3—C16—N2	6.2 (3)	Zn—S2—C38—N6	−1.62 (18)
C17—N3—C16—S1	−172.37 (17)	Zn—S2—C38—N7	176.80 (13)
Zn—S1—C16—N2	−5.44 (18)	C38—N7—C39—C40	−12.1 (3)
Zn—S1—C16—N3	173.01 (14)	C38—N7—C39—C44	164.5 (2)
C16—N3—C17—C18	165.8 (2)	C44—C39—C40—C41	−2.2 (3)
C16—N3—C17—C22	−12.3 (3)	N7—C39—C40—C41	174.3 (2)
C22—C17—C18—C19	1.0 (3)	C39—C40—C41—C42	1.0 (4)
N3—C17—C18—C19	−177.24 (18)	C40—C41—C42—C43	1.2 (4)
C17—C18—C19—C20	0.6 (3)	C41—C42—C43—C44	−2.0 (4)
C18—C19—C20—C21	−1.2 (3)	C42—C43—C44—C39	0.8 (4)
C19—C20—C21—C22	0.1 (4)	C40—C39—C44—C43	1.4 (3)
C20—C21—C22—C17	1.5 (3)	N7—C39—C44—C43	−175.4 (2)

### Hydrogen-bond geometry (Å, º)

**Table:**

*D*—H···*A*	*D*—H	H···*A*	*D*···*A*	*D*—H···*A*
N7—H7*N*···O4^i^	0.87 (2)	2.17 (2)	3.019 (2)	165 (2)
C44—H44···O4^i^	0.95	2.49	3.305 (3)	144
C37—H37···O3^ii^	0.95	2.48	3.373 (3)	157
C14—H14···O1^iii^	0.95	2.54	3.462 (3)	164

Symmetry codes: (i) *x*, *y*−1, *z*; (ii) −*x*+3/2, *y*−1/2, −*z*+1/2; (iii) −*x*+1, −*y*+1, −*z*+1.
